# Left Acetabular Osteoarticular Cryptococcosis in an Immunosuppressed Renal Transplant Recipient: A Case Report and Literature Review

**DOI:** 10.7759/cureus.108033

**Published:** 2026-04-30

**Authors:** Pankhuri Kumari, Rahul Ranjan, Twishi Shrimali, Arupparna Sengupta, Gaurav Khanna, Rahul Tanwar, Kunal Gandhi, Sharmila Sengupta, Sadia Khan

**Affiliations:** 1 Department of Microbiology, Amrita School of Medicine, Faridabad, IND; 2 Department of Pathology, Amrita School of Medicine, Faridabad, IND; 3 Department of Radiology, Amrita School of Medicine, Faridabad, IND; 4 Department of Nephrology, Amrita School of Medicine, Faridabad, IND

**Keywords:** acetabular osteomyelitis, cryptococcus neoformans (c. neoformans), immunosuppression, next-generation sequencing (ngs), post-renal transplant

## Abstract

In individuals with weakened immune systems, opportunistic fungal infections remain a major clinical challenge, particularly among solid organ transplant recipients. A 60‑year‑old post‑renal transplant patient with diabetes and hypertension presented with fever and escalating pain in the left hip. Imaging revealed an osteolytic lesion of the left acetabulum, initially suggestive of osteomyelitis. Fine needle aspiration cytology demonstrated encapsulated yeast forms, and special stains confirmed *Cryptococcus *species. Culture and targeted next‑generation sequencing identified *Cryptococcus neoformans*. The patient was treated with liposomal amphotericin B and high‑dose fluconazole, with therapy modified due to amphotericin‑associated nephrotoxicity. Clinical improvement was achieved following a tailored antifungal regimen and close monitoring of renal function. This case highlights the diagnostic difficulties posed by cryptococcal osteomyelitis in transplant recipients, where musculoskeletal symptoms can mimic bacterial or neoplastic conditions. It emphasizes the role of advanced molecular diagnostics for species confirmation when conventional methods are inconclusive, and the importance of individualized antifungal therapy in patients with renal compromise. Our report contributes to the limited documentation of acetabular involvement and reinforces the need to consider fungal etiologies in immunosuppressed patients presenting with atypical bone lesions.

## Introduction

Invasive fungal infections of opportunistic origin remain an important contributor to illness and death among people with compromised immune defenses. Among these pathogens, *Cryptococcus neoformans* is a notable encapsulated yeast, primarily recognized for causing life-threatening meningoencephalitis [1].

*C. neoformans *infection most frequently occurs in individuals with impaired T-cell-mediated immunity. High-risk groups include those with HIV infection; hematological malignancies such as leukemia, diabetes mellitus, and sarcoidosis; and individuals undergoing corticosteroid therapy. Organ transplant recipients receiving immunosuppressive agents - particularly calcineurin inhibitors and mycophenolate mofetil - are also predisposed, as these medications significantly compromise T cell function, increasing vulnerability to invasive fungal infections, such as cryptococcosis [1].

Cryptococcosis most often affects the lungs and central nervous system, while skeletal involvement is rare. Skeletal cryptococcosis is seen in less than 10% of cases. There are no clinical or radiological features that will indicate skeletal cryptococcosis; hence, it is vital to include it in the differential diagnosis. The vast majority of cases - over 95% - are linked to *C. neoformans*, with the remainder attributed to *Cryptococcus gattii* [2].

## Case presentation

A 60-year-old male visited the Nephrology Outpatient Department with a one-week history of fever and progressively worsening pain in the left hip over the past month. He had undergone an ABO-compatible spousal renal transplant one year ago and was currently receiving immunosuppressive therapy. His medical history included type 2 diabetes mellitus and hypertension. Additionally, he had undergone dorso-lumbar spine fracture fixation in 2017 and dynamic hip screw fixation for a right hip fracture in 2022. Initial plain radiograph of the left hip showed no evidence of arthritis. Magnetic resonance imaging (MRI) done at another hospital revealed changes suggestive of osteomyelitis in the supra-acetabular region.

On examination, vital parameters were stable: temperature of 98.1°F, blood pressure of 130/90 mmHg, pulse of 80/min, and oxygen saturation of 97% on room air. Systemic examination revealed normal cardiovascular findings with regular heart sounds and no audible murmurs. Respiratory assessment showed bilateral air entry without abnormal breath sounds. Abdominal examination found the abdomen to be soft and nontender, with no signs of distension or guarding. The central nervous system evaluation was unremarkable, with no focal neurological deficits noted.

Laboratory evaluation demonstrated normal renal function but revealed suboptimal glycemic control, mild anemia, and elevated inflammatory markers. The detailed results are summarized in Table 1.

**Table 1:** Laboratory findings with reference ranges

Test	Result	Units	Reference Range
Serum Urea	24	mg/dL	9-50
Serum Creatinine	0.79	mg/dL	0.7-1.3
Hemoglobin	11.5	g/dL	13.0-17.0 (male)/12.0-15.0 (female)
HbA1c	7.0	%	0-5.69
C‑Reactive Protein (CRP)	2.11	mg/dL	0-1
WBC Count	6300	cells/uL	4000-11,000
Lymphocyte	20.5	%	20-40%
Neutrophil	64.9	%	40-70%

**Figure 1:**
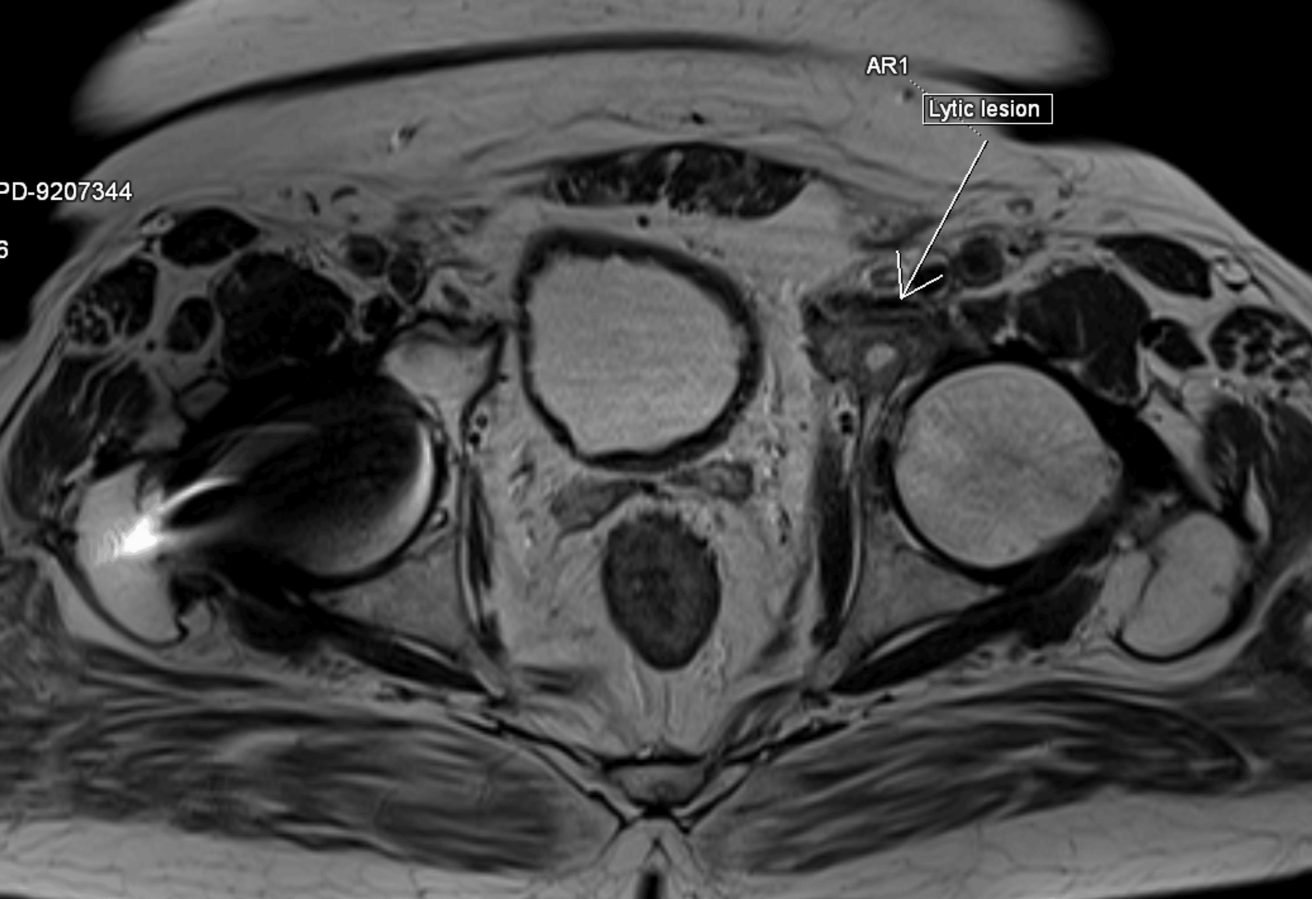
CT-MRI of the pelvis with bilateral hips revealed an osteolytic soft tissue lesion involving the left acetabulum

An orthopedic consultation was obtained for the patient's left hip pain. Based on the evaluation, a contrast-enhanced MRI of the pelvis, including bilateral hips, was advised for further assessment. Contrast-enhanced MRI (Figure 1) of the pelvis with bilateral hips revealed an osteolytic soft tissue lesion involving the left acetabulum, accompanied by adjacent marrow edema. 

The differential diagnosis for this finding includes eosinophilic granuloma, plasmacytoma, and metastasis. The patient was empirically started on IV meropenem in view of suspected osteomyelitis.

Ultrasound-guided aspiration of the left hip joint was performed. Fine needle aspiration cytology (FNAC) of the aspirated lesion demonstrated numerous yeast forms of variable sizes, characterized by narrow-based budding and a refractile, uniformly unstained capsule. These fungal elements were noted both within macrophages and in the extracellular milieu.

**Figure 2:**
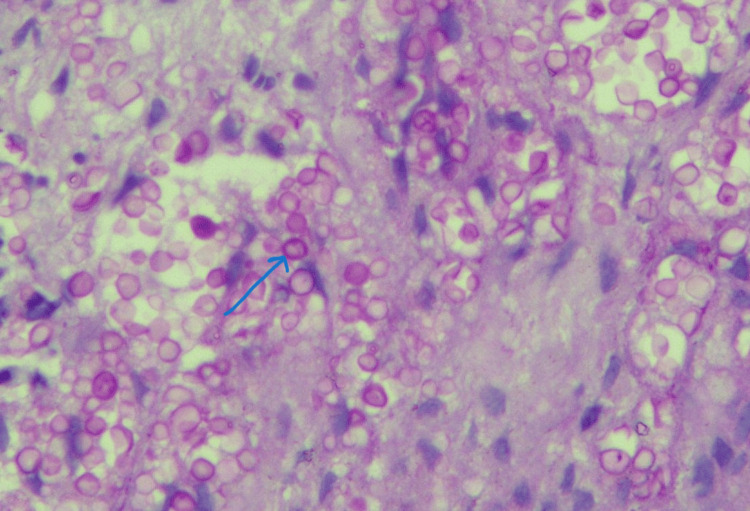
Periodic Acid–Schiff (PAS) stain depicting budding yeasts representing Cryptococcus species

Special stain - periodic acid-Schiff (PAS) (Figure 2) -highlighted fungal profiles morphologically consistent with *Cryptococcus *species.

Additional diagnostic investigations, including bacterial culture, Ziehl-Neelsen (ZN) staining, potassium hydroxide (KOH) preparation for fungal elements, and GeneXpert Ultra for Mycobacterium tuberculosis, all returned negative results. Paired blood cultures from peripheral venipuncture and urine culture were also sterile. Importantly, the serum cryptococcal antigen test was performed and was positive, supporting the diagnosis of cryptococcal infection.

In light of persistent left hip pain and a strong clinical suspicion of underlying bone pathology, a CT-guided bone biopsy was performed targeting the left acetabular region to obtain tissue for definitive diagnosis.

Histopathological examination of Tru-cut needle biopsy sections revealed a linear core of fibrocollagenous tissue with infiltration by chronic inflammatory cells and macrophages. Numerous narrow-based budding spores consistent with *Cryptococcus*, characterized by their thick capsules, were identified within the tissue. There was no histological evidence of neoplasia. PAS effectively highlighted the *Cryptococcus *spores, confirming the fungal etiology.

**Figure 3:**
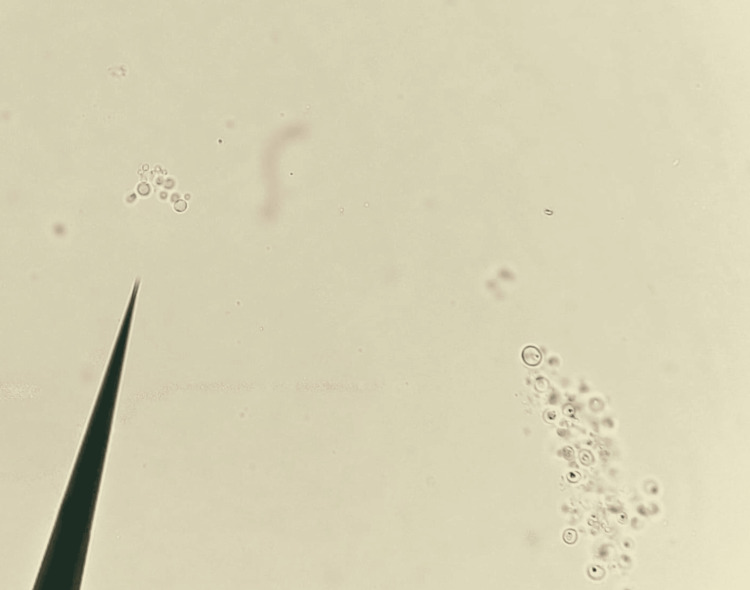
Round budding yeast cells seen in 10% KOH mount of the acetabular tissue KOH: potassium hydroxide

A 10% KOH mount of the tissue showed round budding yeast cells, as shown in Figure 3.

**Figure 4:**
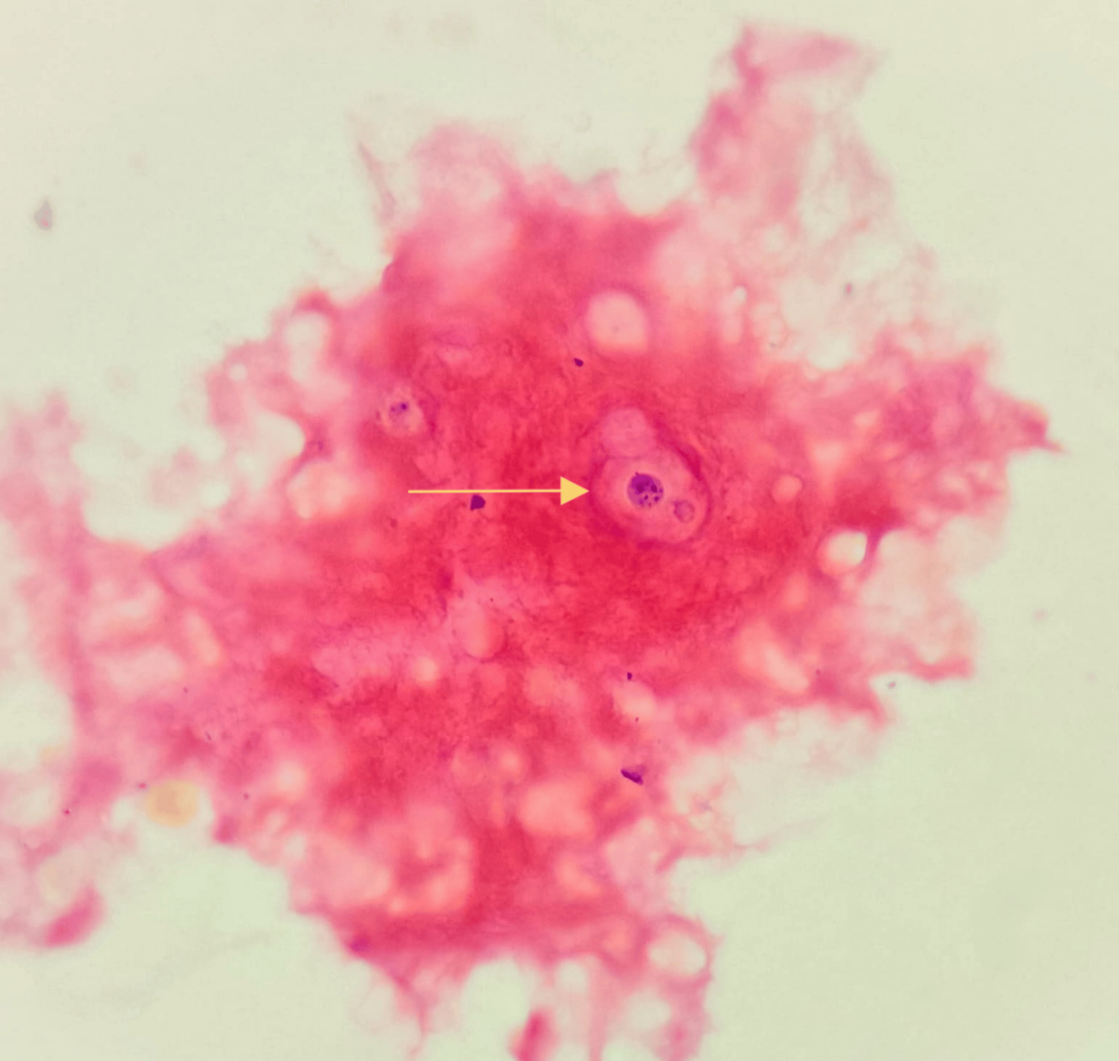
Gram stain of the biopsy tissue showed Gram-positive round budding yeast cells

 Gram stain of the biopsy tissue showed Gram-positive round budding yeast cells, as shown in Figure 4.

**Figure 5:**
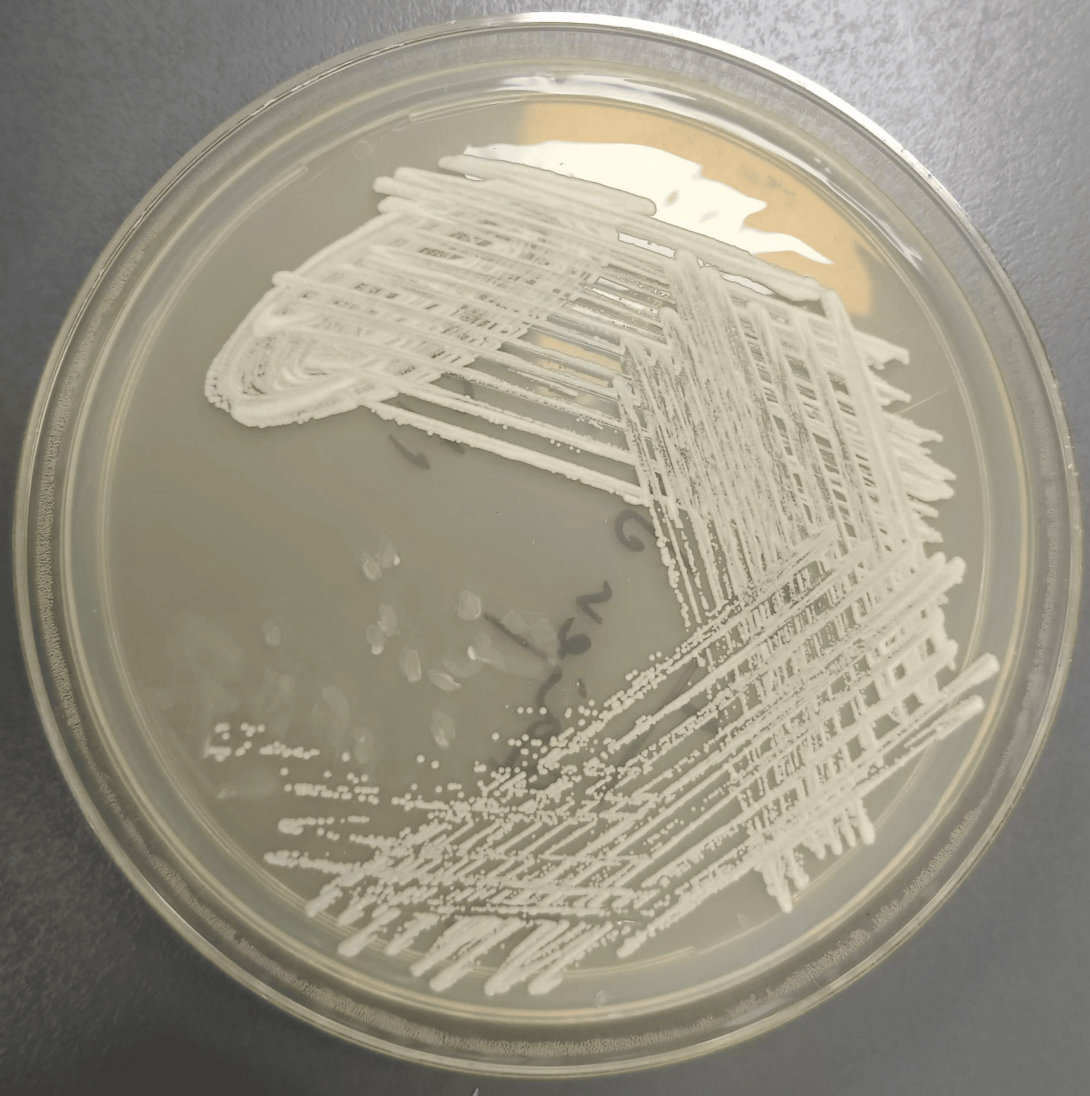
Culture performed on Sabouraud dextrose agar (SDA) grew white, pasty colonies

Culture performed on Sabouraud dextrose agar (SDA) grew white, pasty colonies, as shown in Figure 5. 

The isolate was put for VITEK identification, which identified it as *C. neoformans/C. gattii *with 50% probability. The isolate was susceptible to fluconazole (minimum inhibitory concentration (MIC): 2) and amphotericin B (MIC: 1) and resistant to flucytosine (MIC: 32). For confirmation of the species, the isolate was submitted for targeted next-generation sequencing (NGS) using the Infexn platform (MinION; Library Kit SQK-NBD 114.96), which definitively identified the organism as *C. neoformans*.

After evaluation by the infectious disease team, therapy was initiated with liposomal amphotericin B at 300 mg intravenously once daily, prepared in 500 mL of 5% dextrose, and infused at 100 mL per hour. Additionally, oral fluconazole 800 mg once daily was prescribed as part of the antifungal treatment regimen [3].

He was receiving oral tacrolimus of 1 mg twice daily, mycophenolate sodium of 180 mg twice daily, and prednisolone of 5 mg once daily as part of his immunosuppressive protocol. The patient was also receiving propranolol of 10 mg twice daily, silodosin of 8 mg once daily, atorvastatin of 10 mg once daily, calcium with vitamin D3 of 500 mg once daily, and pantoprazole of 40 mg once daily, all administered orally.

Glycemic control was managed with subcutaneous insulin regular 10-12-12 units before meals and insulin glargine 22 units subcutaneously at bedtime (10 PM) under strict blood glucose monitoring. Based on the clinical presentation, histopathological examination, special staining characteristics, and molecular confirmation via targeted NGS, the patient was diagnosed with cryptococcal osteomyelitis of the left acetabulum.

During hospitalization, the patient's blood sugars and blood pressure were well controlled. The patient had good urine output with stable renal parameters. Therefore, the patient was discharged with the same medication.

At one-week follow-up, the patient demonstrated elevated renal parameters, with a serum creatinine of 3.79 mg/dL and urea of 58 mg/dL, indicative of liposomal amphotericin B-associated acute kidney injury (AKI). In light of this development, liposomal amphotericin B therapy was discontinued after seven days. At the next follow‑up, the patient was advised to continue fluconazole, with a plan to restart liposomal amphotericin B (Ambisome) at a lower dose if renal parameters improved. Subsequently, Ambisome was re‑initiated at 150 mg IV daily, and serial monitoring demonstrated improvement in renal function with serum creatinine stabilizing at 1.6 mg/dL. Under nephrology supervision, the amphotericin B dose was gradually escalated from 150 mg to 200 mg and then to 250 mg. A total of 15 days of amphotericin B therapy was administered following four months of fluconazole treatment. At the most recent follow‑up, the patient reported improvement in the index symptoms and remains clinically stable.

## Discussion

*C. neoformans *is an encapsulated yeast frequently implicated in opportunistic infections, particularly among immunocompromised hosts, such as individuals with HIV, transplant recipients, and patients with malignancies [1]. As HIV management has improved, the burden of cryptococcosis has shifted, with a growing proportion of cases now occurring in non-HIV populations. This trend is especially evident among organ transplant recipients, who face increased susceptibility due to long-term immunosuppressive therapy. This case demonstrates the trend. Population-based studies from the United States indicate that the overall incidence of cryptococcosis among solid organ transplant (SOT) recipients ranges from 0.2% to 4.1% [4]. Cryptococcosis ranks as the third most common invasive fungal infection among transplant recipients, with incidence reported between 4.5% and 33.8%. This highlights its substantial role in contributing to morbidity and mortality within this high‑risk group [5].

While liver, lung, and heart transplant recipients tend to develop cryptococcosis earlier after transplantation and face higher mortality rates, kidney transplant recipients remain a critical at-risk population. This is attributable to the large number of kidney transplants performed, the unpredictable timing of disease onset, and the lack of routine antifungal prophylaxis in this group [4].

*C. neoformans *accounts for over 95% of cryptococcal infections, predominantly affecting immunocompromised individuals. In contrast, *C. gattii*, though less common, is typically associated with infections in immunocompetent hosts [2]. *Cryptococcus *has a cosmopolitan distribution, frequently recovered from environmental reservoirs such as soil contaminated with avian excreta, particularly from pigeons [6].

*Cryptococcus *typically enters the host via the respiratory tract, where it may induce pulmonary complications. Notably, it exhibits a marked neurotropism, often targeting the central nervous system and leading to severe neurological manifestations [2]. Pulmonary and central nervous system involvement are well-established features of cryptococcosis, whereas osseous involvement is relatively rare. It typically arises from a primary lung infection that disseminates hematogenously, or less frequently, through direct traumatic inoculation via the skin. Osseous cryptococcosis accounts for approximately 5-10% of disseminated cases [2,7,8]. Cryptococcal osteomyelitis most often involves the spine, ribs, femur, tibia, clavicle, pelvis, and humerus, with a tendency to affect flat bones and the metaphyseal regions of long bones. Involvement of the acetabulum is particularly rare, with only a handful of cases documented in the literature [9].

Randomized treatment trials specifically addressing SOT recipients are lacking; consequently, current management recommendations are largely extrapolated from evidence derived in populations living with HIV [5]. Except in cases of direct cutaneous inoculation following trauma, extrapulmonary cryptococcosis is, by definition, considered disseminated disease and typically warrants aggressive induction therapy. However, clinical treatment trials specifically addressing non-pulmonary, non-CNS manifestations are lacking. According to the European Confederation of Medical Mycology (ECMM)/International Society for Human and Animal Mycology (ISHAM)/American Society for Microbiology (ASM) global guideline, the management of cryptococcaemia should follow the same therapeutic approach as that recommended for central nervous system disease [5].

International guidelines advise using the most potent fungicidal induction regimen available. The preferred approach is liposomal amphotericin B at 3-4 mg/kg daily in combination with flucytosine 25 mg/kg four times per day for two weeks. This regimen is strongly recommended in high‑income settings, particularly for SOT recipients and for patients who are neither HIV‑positive nor transplant recipients. An alternative regimen involves a single 10 mg/kg dose of liposomal amphotericin B combined with 14 days of flucytosine and fluconazole 1,200 mg daily; however, this approach has only been evaluated in HIV‑infected individuals within low‑income settings. When administering amphotericin B, including its liposomal form, it is essential to ensure adequate pre‑hydration and proactive supplementation of potassium and magnesium to reduce the risk of treatment‑related toxicities. During treatment, close monitoring is essential, with complete blood counts, renal function, and electrolyte levels checked at least every other day to identify nephrotoxicity and disturbances in bone marrow, fluid balance, and electrolytes. Baseline liver function testing is recommended, followed by repeat assessments at least once weekly throughout the course of therapy [5].

Adherence to consolidation and maintenance therapy is essential for optimal outcomes. The recommended consolidation phase consists of fluconazole 400-800 mg daily for eight weeks, with the higher dose favoured in low‑income settings. Maintenance therapy is subsequently continued with fluconazole 200 mg daily for 12 months, or until immune recovery has been achieved. Close attention must be paid to possible drug-drug interactions, with dosage modifications implemented as required. Therapeutic drug monitoring of tacrolimus, cyclosporine, and sirolimus is recommended, as co‑administration with azole antifungals necessitates dose reduction of these immunosuppressants [5].

In line with international guideline recommendations favouring liposomal amphotericin B combined with flucytosine as the preferred fungicidal induction regimen, our patient was started on liposomal amphotericin B 300 mg intravenously once daily, together with oral fluconazole 800 mg daily. While the guideline strongly advises amphotericin B plus flucytosine for two weeks, particularly in SOT recipients and non-HIV populations, flucytosine was not administered in this case due to local practice constraints. Instead, fluconazole was used as adjunctive therapy, consistent with consolidation phase recommendations but at a higher daily dose from the outset. The patient's immunosuppressive regimen included tacrolimus, mycophenolate sodium, and prednisolone, necessitating close monitoring for drug-drug interactions as highlighted in the guideline. During follow-up, liposomal amphotericin B was discontinued after seven days due to acute kidney injury, and therapy was subsequently re‑initiated at a reduced dose under nephrology supervision with gradual escalation as renal function stabilized. In total, 15 days of amphotericin B therapy were completed following four months of fluconazole treatment. Thus, while the therapeutic approach aligned with the principles of the global guideline, modifications were required to balance antifungal efficacy with renal safety in the Indian clinical context.

Our case report underscores several important clinical insights. First, it highlights the necessity of considering fungal etiologies, particularly *C. neoformans*, in immunocompromised patients such as SOT recipients who present with atypical musculoskeletal complaints, as such infections may mimic more common bacterial or neoplastic processes. Second, it demonstrates the utility of advanced molecular diagnostics, such as targeted NGS, for accurate species identification when conventional culture methods are inconclusive or delayed. Finally, it emphasizes the potential for nephrotoxicity with amphotericin B therapy, even in its liposomal formulation, reinforcing the importance of vigilant renal monitoring throughout treatment.

We have summarized a few published cases of osseous cryptococcosis in the last five years in Table 2.

**Table 2:** Summary of various published cases of osseous cryptococcosis in last five years NGS: next-generation sequencing

Study/Place/Year	Associated Risk/Underlying disease	Site of Infection/Clinical presentation	Laboratory Identification	Radiological Findings	Treatment Given	Outcome
Present Case Faridabad, India/2025	Renal transplant one year ago, type 2 diabetes mellitus, and hypertension	Left acetabulum/fever and progressively worsening pain in the left hip	Serum cryptococcal antigen test was positive. Trucut needle biopsy: Numerous narrow-based budding spores consistent with *Cryptococcus*, characterized by their thick capsules. Periodic acid-Schiff (PAS) effectively highlighted the *Cryptococcus *spores. Culture (Vitek 2) and targeted NGS	Contrast-enhanced MRI of the pelvis with bilateral hips revealed an osteolytic soft tissue lesion involving the left acetabulum, accompanied by adjacent marrow edema	IV liposomal amphotericin B at a dose of 300 mg once daily and oral fluconazole 800 mg once daily	Treated and recovered
Lee et al. [2] Taiwan, China/2024	No underlying disease	Progressive neck pain for one month and left side chest pain for one week	Serological testing revealed a positive cryptococcal antigen. Grocott-Gomori methenamine silver (GMS) staining and culture supported the diagnosis, while histopathological examination demonstrated a granulomatous reaction consistent with cryptococcosis	Chest X‑ray showed increased markings in the lower lung fields. Sacral imaging revealed mild L5-S1 disc space narrowing. MRI demonstrated lesions in both iliac bones and the inferior L4 vertebra. CT identified a 0.85‑cm ovoid nodule in the right lower lung and osteolytic changes in the sternum, ribs, and right humeral head	The patient received intravenous amphotericin B at 0.7 mg/kg/day together with oral flucytosine 100 mg/kg/day in four divided doses for a duration of four weeks. Following clinical improvement, the patient was discharged on oral fluconazole 400 mg daily, prescribed for one year	Treated and recovered
Li et al. [6] Fujiyan Province, China/2024	No underlying disease	Presented with progressive distal painless swelling of the left forearm	Puncture biopsy revealed sheets of epithelioid cells with scattered multinucleated giant cells. Special staining: Periodic acid-Schiff stain was consistent with mycosis. Culture and next-generation sequencing (NGS) Antifungal susceptibility was not done	X‑ray revealed osteolytic involvement of the radial diaphysis. CT scan: Showed irregular bone destruction at the distal end of the radius	Oral fluconazole 200 mg BD consolidation therapy for 12 weeks	Treated and recovered
Papadakis et al. [10] Greece, Europe/2023	Diabetic patient with arterial hypertension, chronic kidney disease, hypothyroidism, and rheumatoid arthritis under therapy with methotrexate and corticosteroids	3-day pain located mainly over the left tibia’s anterior surface, accompanied by swelling and redness. She had an ulcerative lesion over the upper outer quarter of her left breast	Hematoxylin and eosin stain, Grocott-Gomori methenamine silver (GMS). Culture was positive from the biopsy of the tibia and breast	Plain radiography of the tibia demonstrated an osteolytic area in the upper third of the diaphysis. By day 3, MRI revealed a bone marrow lesion in the proximal diaphysis, measuring approximately 3 cm in length, with a heterogeneous pathological composition	200 mg fluconazole twice a day for a total duration of 9 months	Treated and recovered
Jia et al. [11] Yibin, China/2023	No underlying disease	Presented with a more than 4-month history of low back pain, pain radiating to the left limb, and left limb numbness	Blood for cryptococcal antigen: Positive. Needle biopsy suggested a cryptococcal lesion	CT scan revealed a lytic lesion involving the left half of the L4 vertebral body with extension into adjacent paravertebral soft tissue. MRI demonstrated a paraspinal soft tissue mass at L4 resembling a tumor.	Oral fluconazole at 400 mg daily was continued for six months following surgery	Treated and recovered
Zhong et al. [12] Ganzhou, China/2023	No underlying disease	Progressive low back and sacrococcygeal pain for 3 months	CT‑guided biopsy specimens stained with hematoxylin and eosin demonstrated granulomatous inflammation with extensive macrophage infiltration. Grocott’s methenamine silver staining revealed numerous small yeast‑like organisms with black staining. Periodic acid-Schiff staining highlighted multiple spheroid structures of varying sizes with red‑stained outer membranes. Culture confirmation was obtained, and species identification was performed using MALDI‑TOF	CT scan showed irregular osteolytic lesions in the sacrum surrounded by multiple cystic, low‑density soft tissue masses of varying size. MRI demonstrated patchy sacral signals, appearing hyperintense on T2WI and hypointense on T1WI	The patient received fluconazole for 12 weeks: 4 weeks intravenously at 400 mg/day, followed by 8 weeks orally at the same dose	Treated and recovered
Ma et al. [13] Guangxi Province, China/2022	No underlying disease. She had surgery for a right olecranon fracture two years ago; the wound healed well, and limb function fully recovered	Pain for 2 months and skin ulceration on her right forearm	Histopathology supported chronic osteomyelitis. Fungal culture	X‑ray revealed multiple irregular osteolytic lesions in the mid‑distal shaft of the ulna with minimal periosteal reaction. CT confirmed the largest lesion in the same region of the right ulna	Postoperatively, the patient received intravenous fluconazole 200 mg twice daily for three days. On the fourth day, antifungal therapy was transitioned to intravenous voriconazole 200 mg twice daily	Treated and recovered

## Conclusions

This case illustrates the rare presentation of cryptococcal osteomyelitis of the acetabulum in a renal transplant recipient, emphasizing the diagnostic complexity of musculoskeletal fungal infections in immunocompromised patients. This case underscores the need to maintain a high index of suspicion for fungal etiologies when assessing atypical bone lesions, particularly in transplant recipients, where bacterial or neoplastic causes are often considered first. Advanced molecular diagnostics, such as targeted next‑generation sequencing, proved invaluable for definitive species identification when conventional methods were inconclusive. Furthermore, the therapeutic journey underscores the delicate balance between antifungal efficacy and drug‑related toxicity, especially in patients with pre‑existing renal compromise. Ultimately, individualized treatment strategies, vigilant monitoring, and interdisciplinary collaboration were key to achieving clinical stability. This case expands the scarce evidence base on acetabular cryptococcosis and underscores the critical need for greater clinical awareness of opportunistic fungal infections in solid organ transplant recipients.
